# Preparation of alginate–chitosan–cyclodextrin micro- and nanoparticles loaded with anti-tuberculosis compounds

**DOI:** 10.3762/bjnano.7.112

**Published:** 2016-08-24

**Authors:** Albert Ivancic, Fliur Macaev, Fatma Aksakal, Veaceslav Boldescu, Serghei Pogrebnoi, Gheorghe Duca

**Affiliations:** 1 Laboratory of Organic Synthesis and Biopharmaceutics, Institute of Chemistry of ASM, Academiei 3, MD-2028 Chisinau, Moldova; 2 Department of Chemistry, Faculty of Science, Gebze Technical University, Kocaeli, 41400, Turkey

**Keywords:** chitosan, β-cyclodextrin, density functional theory (DFT), isoconazole, isoniazid, molecular docking, quaternary system, sodium alginate

## Abstract

This paper describes the synthesis and application of alginate–chitosan–cyclodextrin micro- and nanoparticulate systems loaded with isoniazid (INH) and isoconazole nitrate (ISN) as antimycobacterial compounds. Preparation and morphology of the obtained particles, as well as antimycobacterial activity data of the obtained systems are presented. Docking of isoconazole into the active site of enoyl–acyl carrier protein reductase (InhA) of *Mycobacetrium tuberculosis* was carried out in order to predict the binding affinity and non-covalent interactions stabilizing the InhA–isoconazole complex. To assess these interactions, frontier molecular orbital calculations were performed for the active site of InhA and isoconazole obtained from docking. Isoconazole was predicted to be an active inhibitor of InhA with the analysis of the molecular docking and electron density distribution. It has been detected that alginate–chitosan–cyclodextrin microparticulate systems loaded with INH and ISN are as effective as pure INH applied in higher dosages.

## Introduction

Tuberculosis, together with HIV infection, is a leading cause of death with 1.5 million deaths in 2014 worldwide [1]. The biggest challenges regarding a successful treatment of tuberculosis infections are the necessary high dosages and various side effects of the majority of the existent antituberculosis drugs, a long duration of treatment, repellent organoleptic properties, and the high frequency of administration [1–2]. All of these often cause reduced compliance of the patient with the treatment regimen. The last factor, alongside with the low quality of some antituberculosis drugs (insufficient enantiomeric purity) and reduced bioavailability, cause the development of drug-resistant (DR-TB), multidrug-resistant (MD-RTB), and extensively drug-resistant (XDR-TB) tuberculosis [1–2].

In order to reduce the duration of treatment and the frequency and quantity of the administered drugs, and to avoid first-pass effects and to reduce the side effects, various different micro- and nanoparticle-based alginate–chitosane–cyclodextrin systems loaded with antituberculosis drugs for nebulisation have been proposed. Similar systems have been shown effective for the delivery of a celecoxib–hydroxypropyl-β-cyclodextrin–PVP complex [3] and for the controlled release of insulin after oral delivery [4–5]. Since these were mainly administered through the digestive tract, their sizes were only determined by bioavailability, and not by aerosol stability as in the case of inhalational delivery. This fact enabled the authors to synthesize systems with sizes lower than 500 nm, which are not possible if high aerosol stability and compositional stability are important. The application of aerosol-based pharmaceutical compositions has certain advantages in the treatment of lung infections. Among others, these are the reduction of the systemic toxicity, the availability of high drug concentrations at the site of action, and the avoidance of the first-pass effect [6]. The main challenges connected to this type of formulations are problems with correct dosage, enzymatic degradation of the active compound in the lungs and the high cost of production. These challenges can be addressed with the formulation discussed in this article.

Previously, advantages of similar formulations have been discussed by different research groups [7–9]. These include:

• high surface-area-to-volume ratio of the particles [8] leading to a high rate of dissolution and absorption;

• high potential of the microparticles for penetrating the target cells (alveolar macrophages in the case of *M. tuberculosis* lung infection) [10];

• the ability to maintain a high concentration of the active compound at the site of infection for a longer time, which consequently reduces the frequency of drug administration [10];

• interaction with plasma proteins [11], which has been shown to influence the biokinetics of the particles [12].

The main challenges in the usage of aerosols with microparticles for inhalation are connected to the loss of the compound during the inhalation. This problem can be resolved via maintaining particle sizes in the range of 0.5–5.0 μm. Particles with diameters below 0.5 μm are mostly lost at exhalation, because they produce a stable aerosol, which does not precipitate in the lungs. At the same time, particles with diameters above 5.0 μm are mainly retained in the oropharynx [13].

The addition of cyclodextrins to the microparticles results in (i) an increase of stability, solubility, and bioavailability of the active compounds in the complex; this has been proved for the delivery of antifungal triazole and imidazole compounds [14–15]; and (ii) an increased penetration of cell walls. There have been numerous examples in the literature about this [2,15–16]. In the case of *M. tuberculosis*, cyclodextrins extract the cholesterol deposited in the bacterial cell walls [17], disorganizing their double lipid layer and increasing their penetrability for active compounds [18].

**Figure 1:**
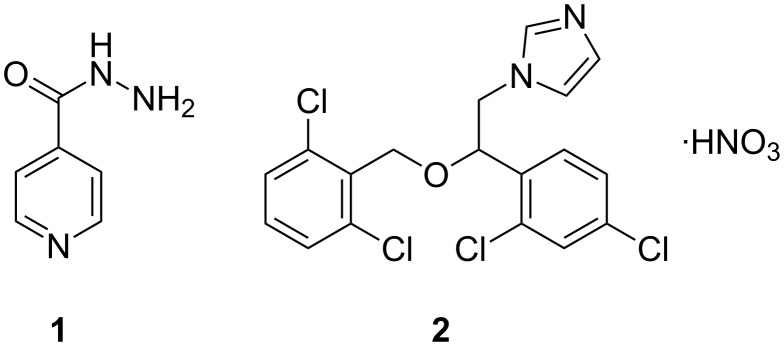
Molecular structures of INH (**1**) and ISN (**2**).

We propose the preparation of chitosane–alginate–cyclodextrin particles containing the antimycobacterial compounds isoniazid (INH) and isoconazole nitrate (ISN). INH (4-pyridinecarboxylic acid hydrazide, Figure 1, compound **1**) is a white crystalline powder soluble in water, slightly soluble in ethanol, chloroform and hardly soluble in ether. INH has antimycobacterial properties and is a first-line agent in the treatment of pulmonary and extrapulmonary tuberculosis [19]. ISN (1-[2-(2,4-dichlorophenyl)-2-[(2,6-dichlorophenyl)methoxy]ethyl]-1*H*-imidazole mononitrate, Figure 1, compound **2**) is a white to light yellow powder soluble in methanol, dimethyl sulfoxide, slightly soluble in ethanol and hardly soluble in water. ISN has antifungal activity and is used to treat dermatophytosis and candidosis (cutaneous and vaginal) [20].

The main mechanism of the fungistatic effect of ISN is the inhibition of lanosterol 14a-demethylation in the ergosterol biosynthesis in the fungal membrane. At the same time, in the case of a prolonged use of ISN, a fungicidal effect can be observed, which is unrelated to the inhibition of the ergosterol synthesis but rather involves rapid membrane damage. As antimycotic, ISN is active against dermatophytes, moulds, yeast, and yeast-like fungi. It is also active against *Corynebacterium minutissimum*, the microorganism causing erythrasma [21–22]. In addition to this, ISN has an antibacterial effect against gram-positive bacteria (*B. cereus, C. tuberculostearicum*, *S. aureus MR, S. haemolyticus, S. hominis,* and *S. salivarius*) confirmed by numerous reports on its antibacterial activity [21–25]. The antibacterial activity of ISN has been suggested to be a result of the binding of isoconazole to oxidized flavohaemoglobin, which results in a decomposition of the enzyme-produced superoxide, a common indicator of cellular stress [21].

ISN, as well as other imidazole antifungals, have been demonstrated to inhibit testicular 17α-hydroxylase and 17,20-lyase and have been suggested to be useful in clinical situations requiring the suppression of androgen production, such as the treatment of hormone-dependent prostatic cancer [26]. Moreover, this group has been discovered to be inhibitors of aromatase, 4-hydroxyandrostenedione and aminoglutethimide [27].

We have suggested that isoconazole is an active inhibitor of enoyl–acyl carrier protein reductase (InhA) from *M. tuberculosis*. InhA is one of the key enzymes involved in the fatty acid biosynthesis of the mycobacterium and is an effective antimicrobial target. InhA inhibitors are promising candidates for the development of novel antitubercular agents [28]. In this study, docking of isoconazole into the active site of InhA was carried out to predict the binding affinity and non-covalent interactions between them. Density functional theory (DFT) based calculations were also performed for the active site of InhA and isoconazole in order to discuss these interactions with the frontier molecular orbital (FMO) analysis of the electron density distribution.

The main carriers used in our particles are β-cyclodextrin, sodium alginate, and chitosan. β-Cyclodextrin (β-CD), a cyclic oligosaccharide consisting of seven D-glucopyranonsyl units. Because of its size and specific structure it can form inclusion complexes with preferentially lipophilic low-molecular-weight compounds. Sodium alginate is a salt of alginic acid, a naturally occurring linear polysaccharide consisting of 1,4-linked β-D-mannuronate and α-L-guluronate units [29]. Sodium alginate forms gels in the presence of certain bivalent cations, particularly calcium ions, and entrap other materials in the formed gel structures [30]. Chitosan is a naturally occurring aminopolysaccharide [31] consisting of randomly distributed of β-1,4-linked D-glucosamine and *N*-acetyl-D-glucosamine units. Chitosan is mainly used as carrier for different pharmaceutical compositions and also shows some antimicrobial activity [31–32]. These three saccharides are practically non-toxic, biocompatible and biodegradable.

The aim of the paper is to obtain and analyze the quaternary systems (systems 1–5): ISN/INH–β-cyclodextrin–alginate–chitosan with potentially enhanced antimycobacterial activity and bioavailability, and low toxicity.

## Results and Discussion

### Morphology of microparticulate systems

**Figure 2:**
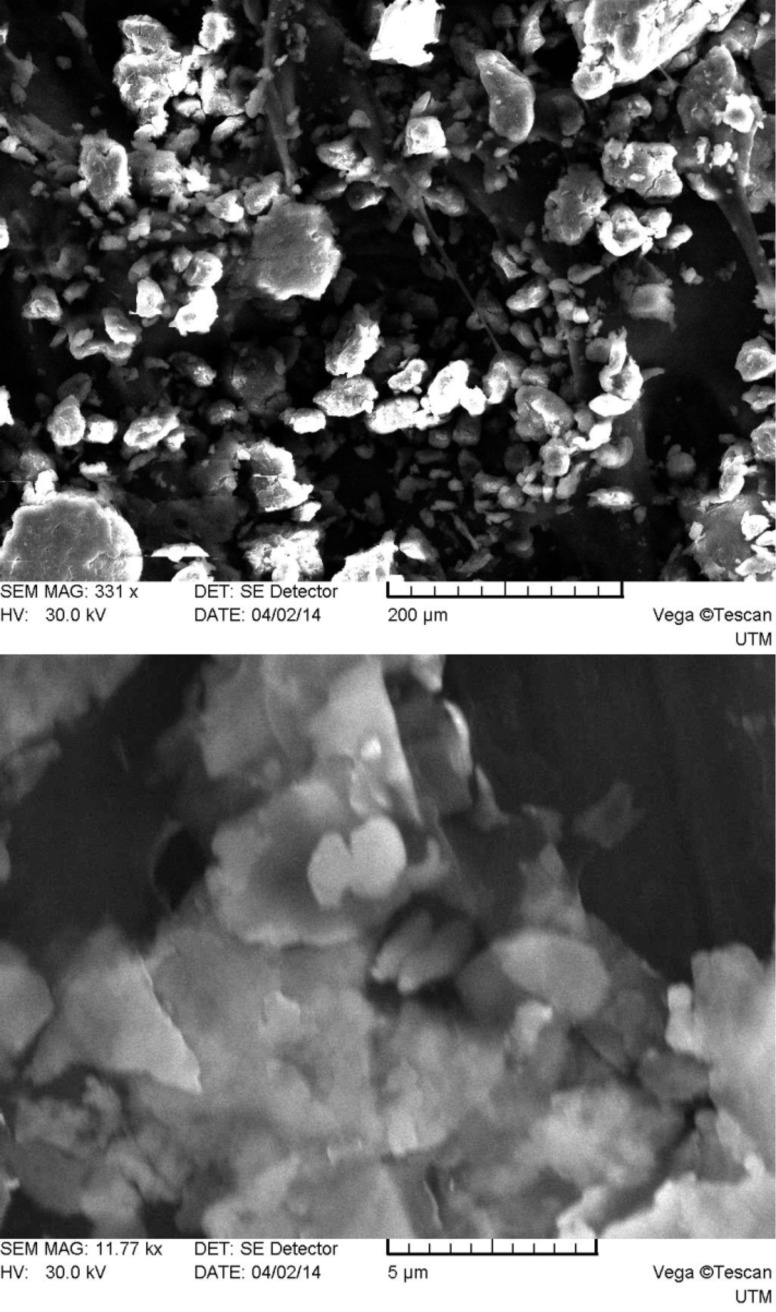
SEM micrographs of system 1 particles (top: magnification 331×, bottom: 11770×).

**Figure 3:**
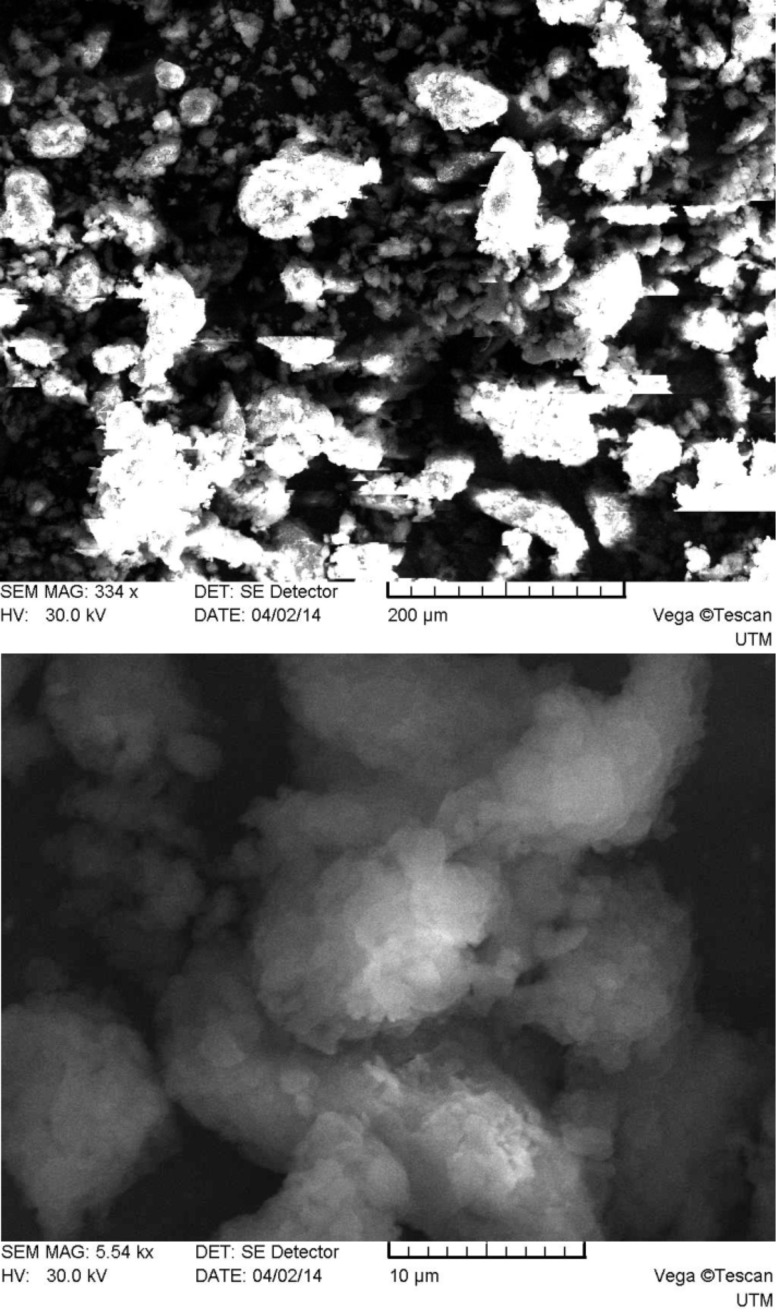
SEM micrographs of system 2 particles (top: magnification 334×, bottom: 5540×).

**Figure 4:**
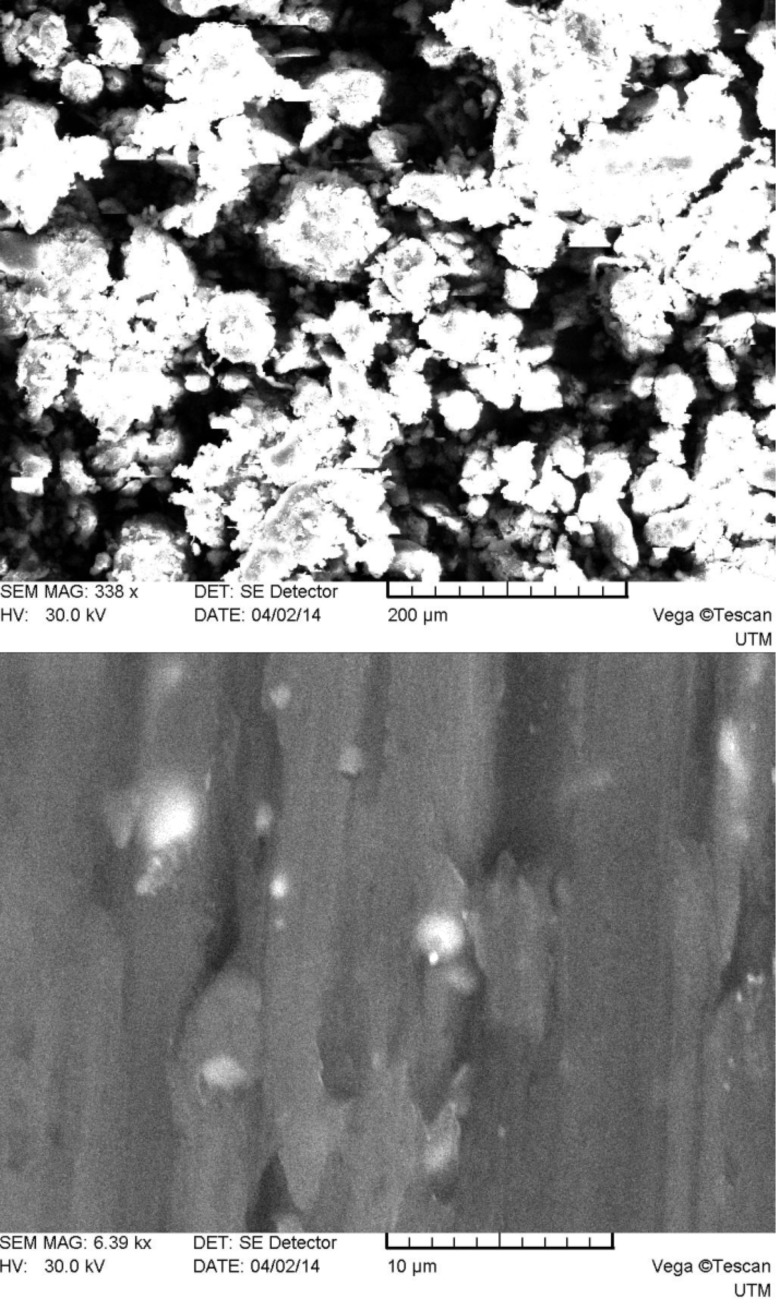
SEM micrographs of system 3 particles (top: magnification 338×, bottom: 6390×).

**Figure 5:**
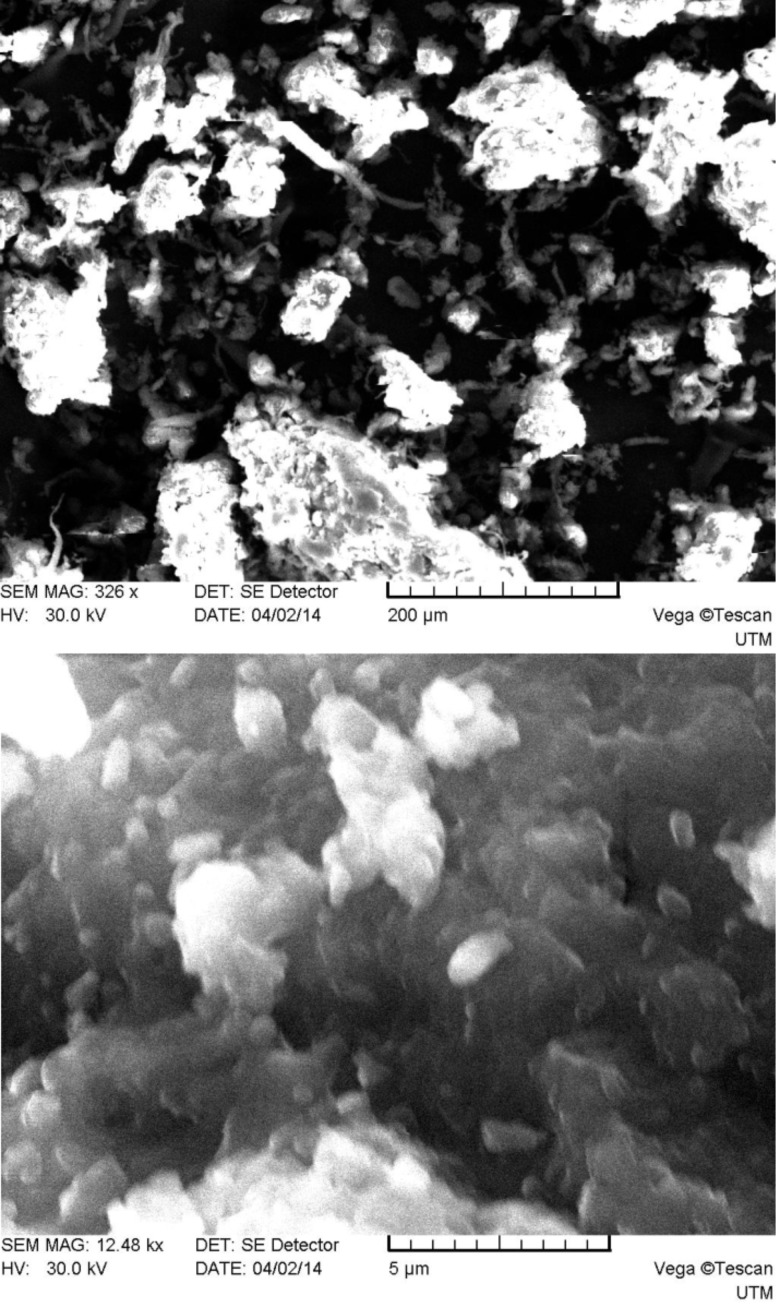
SEM micrographs of system 4 particles (top: magnification 326×, bottom: 12480×).

**Figure 6:**
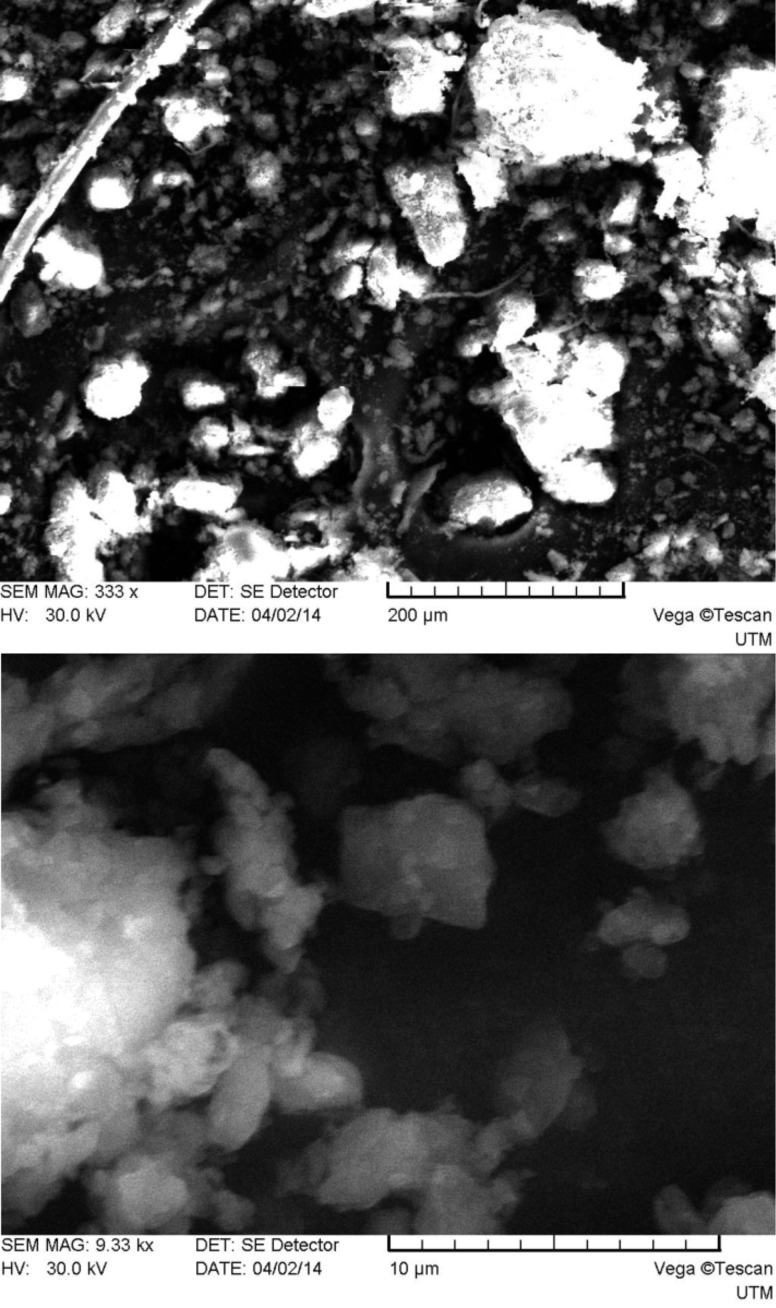
SEM micrographs of system 5 particles (top: magnification 333×, bottom: 9330×).

As one can see from Figure 2, system 1 consists of a mixture of irregularly shaped micro- and nanoparticles with predominance of the first. The main size range of the obtained particles falls in the limits of approximately 0.1–250 μm. The particles surface is smooth compared to those of systems 2–5 (see below Figures 3–6), which have many rough edges, probably due to the excess of β-cyclodextrin that adhered to the surface of alginate–chitosan matrix. The particles of systems 2, 3 and 5, compared to systems 1 and 4, are almost spherical. System 4 contains filamentous formations, characteristic for calcium alginate. Particles with sizes below 100 nm could not be detected because the obtained images were rather unclear.

**Figure 7:**
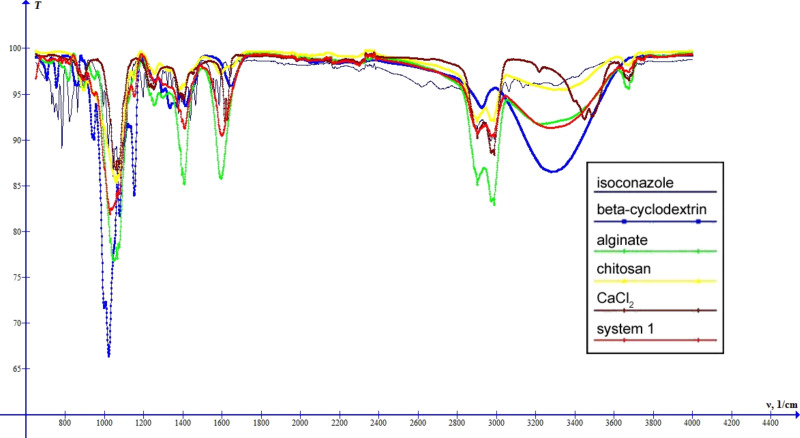
IR spectra of ISN, β-cyclodextrin, sodium alginate, chitosan, calcium chloride and system 1 (ISN–β-cyclodextrin–alginate–chitosan).

Further information was obtained from the analysis of the FTIR spectra. As one can see from Figure 7, the spectrum of system 1 is mainly the sum of the spectra of its components. However, some of the absorption bands of the compounds disappear in the spectrum of the system. These are, for instance, the bands at 754 cm^−1^ and 2924 cm^−1^ in the β-CD spectrum, which correspond to the vibrations (δCCO + δCCH) and ν_as_CH, respectively, and the bands at 862 cm^−1^, 1439 cm^−1^ and 1585 cm^−1^ (C=N bond stretching) in the isoconazole spectrum. Also, one can observe a shift of characteristic band for νOH in the system spectrum (3275 cm^−1^) compared to the respective band of β-CD (3281 cm^−1^). All these observations have led us to conclude that the cyclodextrin and isoconazole molecules are incorporated into the alginate–chitosan structures and that there are interaction forces between the compounds as a result of the formation of the inclusion complex. The absorption band at 3674 cm^−1^ indicates the presence of water molecules in the system.

**Figure 8:**
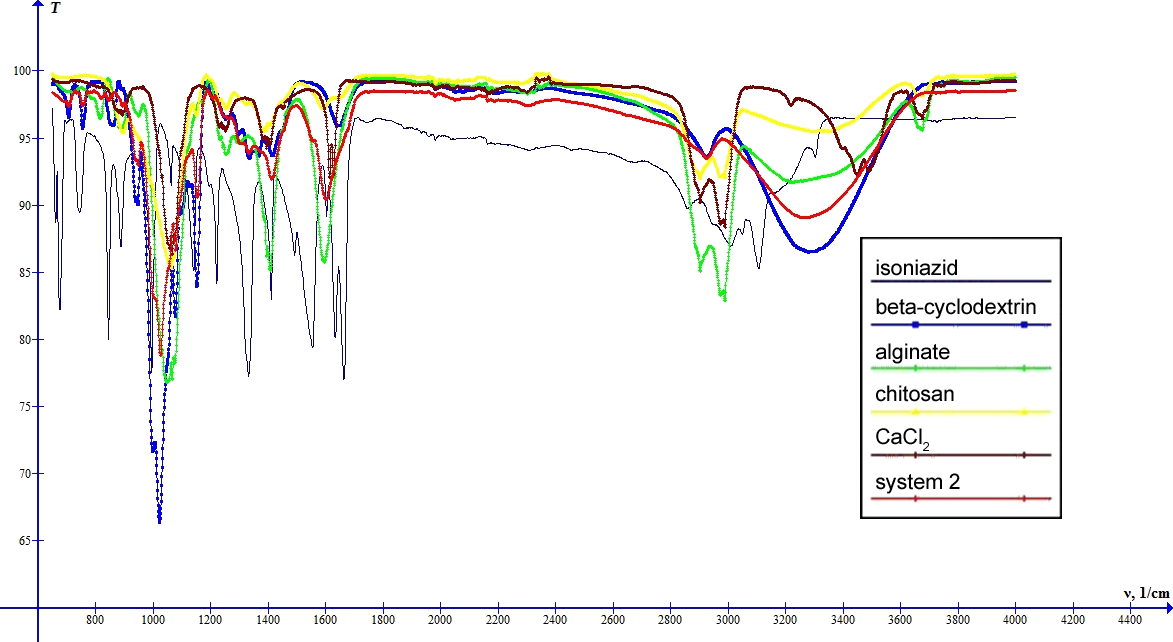
IR spectra of INH, β-cyclodextrin, sodium alginate, chitosan, calcium chloride and system 2.

As seen from Figure 8, in the spectrum of system 2 various bands of INH are shifted or reduced in intensity. These are: 660 cm^−1^ (C–C–C bending), 675 cm^−1^ (C–C=O bending), 743 cm^−1^ (ring C–C–C asymmetric bending), 844 cm^−1^ (ring C–C–H symmetric bending), 887 cm^−1^ (ring C–N–C bending), 1140 cm^−1^ (H–N–H stretching), 1221 cm^−1^ (ring C–C–H asymmetric bending), 1410 cm^−1^ (ring C=C symmetric stretching), 1492 cm^−1^ (C–N–H bending), 1555 cm^−1^ (ring C=N symmetric stretching), 1633 cm^−1^ (ring C=N asymmetric stretching), 2858 cm^−1^ (C-H asymmetric stretching), 3006 cm^−1^ (C–H symmetric stretching) and 3303 cm^−1^ (N-H stretching). This suggests that INH molecules are entirely incorporated in the polymer system. The band at 815 cm^−1^ characteristic for O–Na bonds (in the spectrum of sodium alginate) shifts to 820 cm^−1^ and decreases its intensity in the spectrum of system 2. Also we observe the shifts of the symmetric stretching band of the COO^−^ group of alginate from 1407 cm^−1^ to 1413 cm^−1^, and of the asymmetric stretching band of the COO^−^ group from 1595 cm^−1^ to 1604 cm^−1^. This leads to the conclusion that alginate ions interacted with calcium ions. Also we observe a shift of the band characteristic for OH groups: 3265 cm^−1^ for system 2, compared to 3279 cm^−1^ for β-CD, 3226 cm^−1^ for sodium alginate and 3289 cm^−1^ for chitosan, which demonstrates, probably, the presence of the hydrogen bonds within the obtained system.

**Figure 9:**
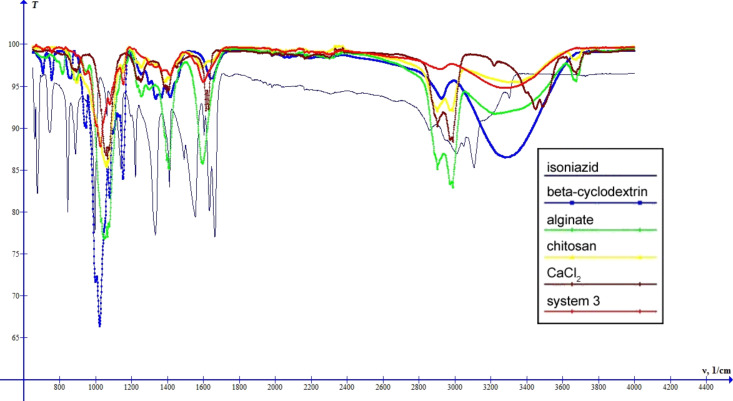
IR spectra of INH, β-cyclodextrin, sodium alginate, chitosan, calcium chloride and system 3.

**Figure 10:**
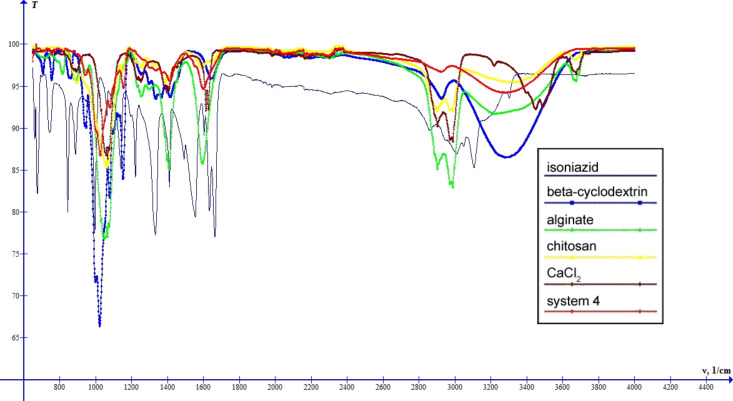
IR spectra of INH, β-cyclodextrin, sodium alginate, chitosan, calcium chloride and system 4.

**Figure 11:**
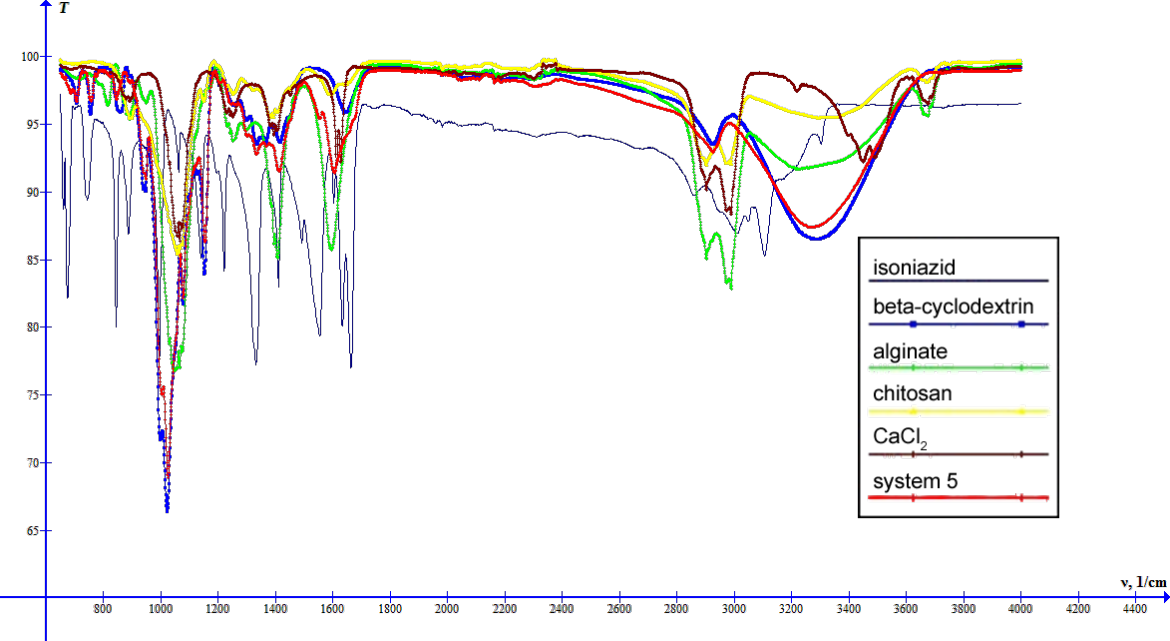
IR spectra of INH, β-cyclodextrin, sodium alginate, chitosan, calcium chloride and system 5.

The spectra of systems 3–5 (Figure 9, Figure 10, Figure 11) are similar to the spectrum of system 2. The differences are shifts of the band characteristic for OH groups: system 3 – 3278 cm^−1^, system 4 – 3277 cm^−1^, system 5 – 3269 cm^−1^, and changes in the intensity of this band. Thus, systems 2–5 can be arranged in the order of increasing intensity of the band characteristic for OH groups: system 3 < system 4 < system 2 < system 5, which suggests an increase of the intensity of interactions between the components of the systems.

### Antimycobacterial activity of isoconazole and microparticulate systems

It has been shown that the use of alginate–chitosan complexes for the administration of azoles in the treatment of experimental tuberculosis is preferable over the use of only cyclodextrin complexes [33]. Therefore, we have proposed complex systems based on alginate–chitosan–cyclodextrin containing INH or isoconazole. Azole antifungals (e.g., econazole, clotrimazole) exhibit a high activity against *M. tuberculosis H37Rv* [34–35]. Our data has shown that ISN also possesses antimycobacterial activity against *M. tuberculosis H37Rv*. This was tested in comparison to rifampicin according to the method described in [36] and given in short in the Methods subsection of the Experimental section.

When encapsulated in the alginate–chitosan–cyclodextrin systems, ISN and INH exhibit a good level of activity at lower concentrations. System 1, producing an isoconazole concentration of 10 μg/mL in the treatment medium, caused 100% inhibition of the growth of *M. tuberculosis*. Comparable results were achieved with the INH-containing microparticulate systems (systems 2–5) at a concentration of active compound of 5–10 μg/mL and with pure INH at a concentration of 100 μg/mL in the treatment medium. We have suggested that the antimycobacterial activity of ISN is the result of its inhibitory action against the enzyme InhA. To confirm our suggestion, we carried out a molecular docking analysis of the isoconazole–InhA interaction.

### Molecular docking results

**Figure 12:**
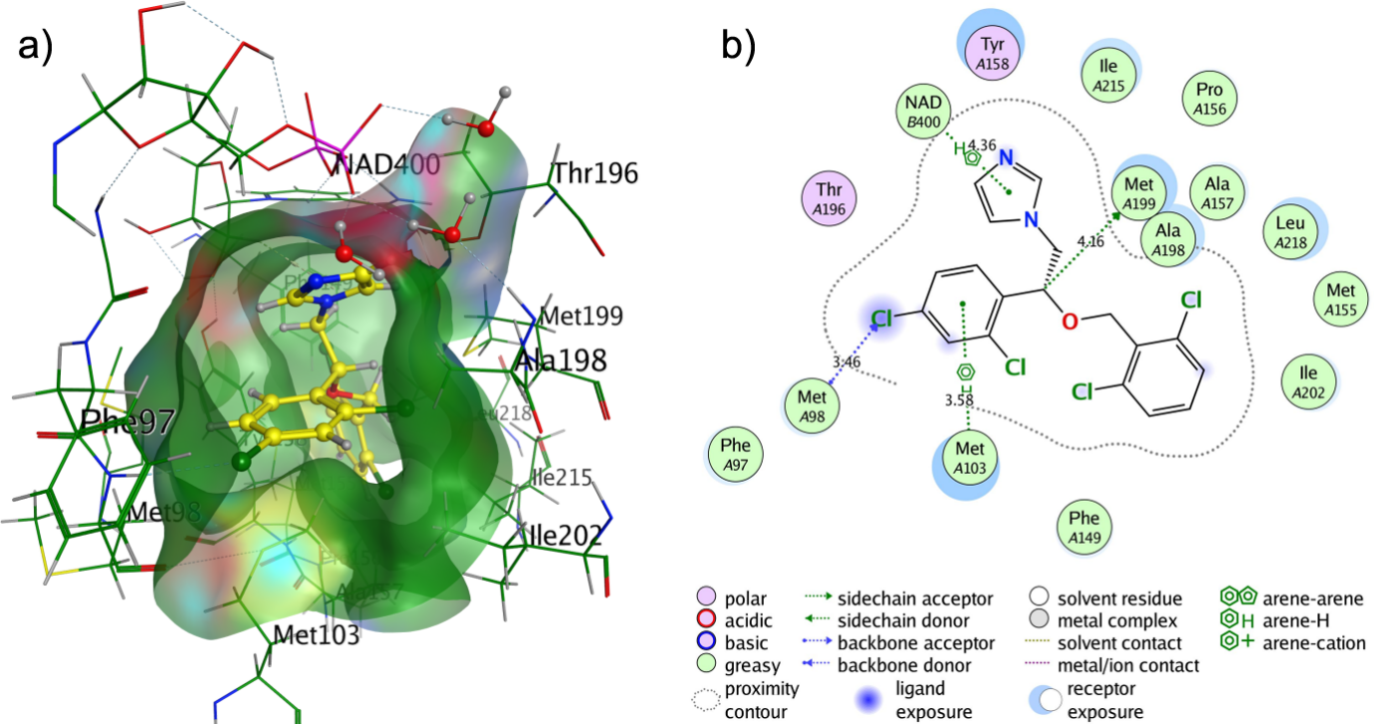
3D (left) and 2D (right) representation of the isoconazole docking pose in the active site of InhA (PDB code: 4U0J).

Docking of isoconazole to enzyme InhA has been carried out in order to predict the binding affinity and non-covalent interactions between them. In Figure 12, the energetically most favourable pose of isoconazole in the active site of InhA is presented in a three-dimensional (3D) and two-dimensional (2D) view.

Isoconazole was located in the active site cavity of InhA with a binding affinity value of −8.2 kcal·mol^−1^ (Figure 12a). As seen in Figure 12b, the interaction of isoconazole with the active site of InhA is dominated by hydrogen bonds. H-bond interactions were predicted between the isoconazole and sulfur-containing methionine amino acid residues of InhA (Met98, Met103, and Met199). Also an arene–H interaction was obtained between the imidazole ring of isoconazole and NAD+ cofactor. The catalytic residues of InhA Tyr158 and Thr196 provided polar interactions with isoconazole. Multiple hydrophobic contacts of amino acids with the inhibitor were indicated by green spheres in Figure 12b.

### Frontier molecular orbital analyses

**Figure 13:**
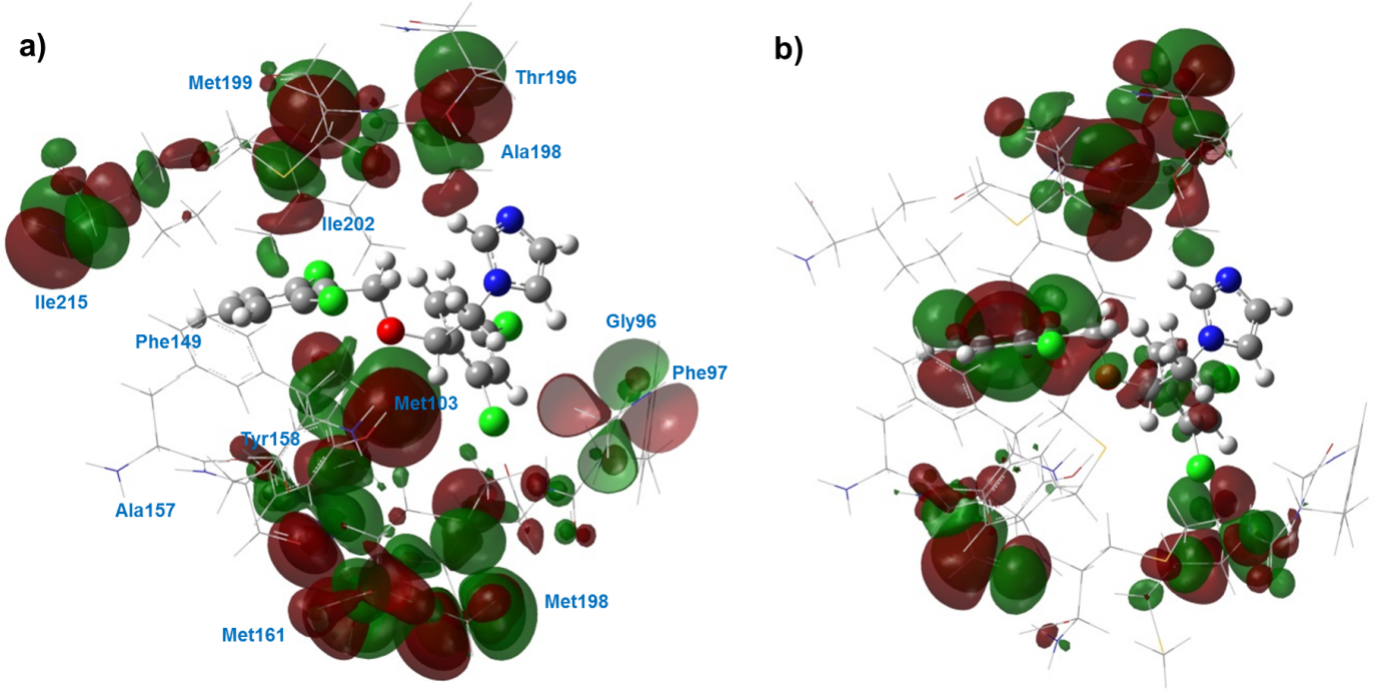
3D view of HOMO (a) and LUMO (b) for the InhA active site interacting with isoconazole.

The analysis of specificity of the enzyme–ligand interaction is closely related to the analysis of frontier orbitals (HOMO and LUMO) in molecular systems. The electron density distribution in the frontier orbitals of the enzyme–ligand complexes under study provides information about the donor–acceptor character of the interactions inside the complexes. The electronic structure calculations were carried out with Gaussian 09 using DFT at the B3LYP/6-31G (d,p) level of theory. The 3D structure of isoconazole and the InhA binding site was taken from the docking calculations. The electron density distribution on the frontier orbitals formed by the active-site residues of InhA with isoconazole is shown in Figure 13.

As seen in Figure 13a, the electron density of the HOMO was distributed on the atoms of the amino acid residues. Methionine amino acids and Ile215, Tyr158, Gly96, and Phe97 residues are involved in the acceptor interaction of the HOMO. In contrast, the role of frontier orbitals in the ligand–receptor interaction was significant for the LUMO (Figure 13b). Atoms of both isoconazole and amino acid residues (Met199, Thr196, Ala198, Ile202, Met161, and Tyr158) contributed to the donor–acceptor interaction.

## Conclusion

The obtained microparticulate and nanoparticulate systems of alginate–chitosan–β-cyclodextrin with isoniazid or isoconazole, which produced media concentrations of active compounds of 5–10 mg/mL, proved to be as effective as pure isoniazid at 100 mg/mL against *M. tuberculosis H37Rv* in culture. The morphology of the obtained particles varies from spherical to irregularly-shaped with sizes ranging from 0.1 to 250 μm. The FTIR spectra analysis revealed molecular interactions between active compounds and the cyclodextrin–polymer moieties of the particles. Isoconazole was predicted as an active inhibitor of InhA after the analysis of the molecular docking and electron density distribution.

## Experimental

### Materials

Chitosan (batch MKBP1333V), alginic acid sodium salt (batch MKBP7317V) were purchased from Sigma-Aldrich Chemie GmbH (Germany); β-cyclodextrin (batch CYL-3190) was purchased from CycloLab R&D Ltd. (Hungary); anhydrous calcium chloride (94–98%) was purchased from Global-Kaustik LLC (Russia); ISN and INH were synthesized by Macaev et al. in 2014 according to the procedure described in [37], at the Institute of Chemistry of the Academy of Sciences of Moldova.

### Methods

#### Bacterial strains and growth conditions

The procedure was adapted from [36]. Shortly, for the first three experiments, *M. tuberculosis H37Rv* inocula were first passaged in radiometric 7H12 broth (BACTEC 12B) until the growth index (GI) reached 800–999. For the fourth replicate experiment, H37Rv was grown in 100 mL of Middlebrook 7H9 broth supplemented with 0.2% glycerol, 10% OADC (oleic acid, albumin, dextrose, catalase), and 0.05% Tween 80 (Sigma), all taken in v/v concentrations. Cultures were incubated in 500 mL nephelometer culture flasks on a rotary shaker at 150 rpm and 37 °C until they reached an optical density of 0.4–0.5 at 550 nm. The, the bacteria were washed and suspended in 20 mL PBS and passed through an 8 mm pore-size filter to eliminate clumps. The filtrates were aliquoted, stored at 280 K, and used within 30 days.

#### Radiometric susceptibility test

For the radiometric susceptibility test, 1/10 mL of BACTEC 12B-passaged inoculum was introduced into 4 mL of test medium without being diluted. Two-fold drug dilutions were prepared in DMSO and introduced with a 0.5 mL insulin syringe in a volume of 50 mL. Frozen inocula were subjected to 1:20 dilution in BACTEC 12B medium, and then 0.1 mL was introduced into the test medium, which yielded 5.0 × 10^5^ CFU per BACTEC vial. Drug-free control vials were composed to include only solvent with bacterial inoculum and solvent with a 1:100 dilution of bacterial inoculum (1:100 controls). All vials were incubated at 37 °C. The GI was determined in a BACTEC 460 instrument until the GI of the 1:100 controls reached at least 30. The next day, all vials were read. The GI and daily change in GI (DGI) were recorded for each drug dilution. The MIC was defined as the lowest concentration for which the DGI value was smaller than the DGI value of the 1:100 control (10). If the GI of the test sample was greater than 100, the sample was considered resistant even if the DGI was less than the DGI of the 1:100 control.

#### Preparation of the microparticulate systems

The quaternary system: ISN–β-cyclodextrin–alginate–chitosan (system 1) was obtained using kneading, the mass ratio of components was 1:2.36:4:4. At the first stage, the binary system ISN–β-cyclodextrin was obtained by kneading, the molar ratio of the components was 1:1, and the working temperature was 20 ± 2 °C. In an agate mortar appropriate amounts of β-cyclodextrin and ISN, previously weighed on the electronic analytical balance model BEL M503i, were added. To the mixture, a sufficient amount of distilled water to form a paste was added. The paste was kneaded with a pestle for 90 min: In the first 60 min by adding distilled water to compensate its loss by evaporation and maintain the appearance of paste. In the next 30 min the mixture was milled to a fine powder. The obtained powder was stored in a sealed tube with parafilm sample, at room temperature (20 ± 2 °C).

A weighed quantity of obtained powder (binary system ISN–β-cyclodextrin) was transferred in a mortar. Then, chitosan according to the proportion 1:2.36:4:4 was added to it, mixed with the pestle, after which distilled water was added till a paste was formed. The obtained paste was kneaded for 30 min and the corresponding amount of sodium alginate was added. The resulting mixture was milled and then 20 mM calcium chloride solution was added to form paste. The paste was milled for 30 min to a fine powder, which was stored in a tube sealed with parafilm, at room temperature (20 ± 2 °C). The mass ratio of CaCl_2_ (solid)/sodium alginate was 1:34.48.

Using the same method, four quaternary systems with INH were prepared: INH–β-cyclodextrin–alginate–chitosan with the following mass ratio of components: 1) 1:8.28:5:5 (system 2); 2) 1:8.28:5:10 (system 3); 3) 1:8.28:10:5 (system 4) and 4) 1:8.28:2.5:2.5 (system 5). The mass ratio of CaCl_2_ (solid)/sodium alginate was 1:10 for systems 2–5.

#### Analysis of obtained systems: morphology of particles, FTIR analysis

The morphology of the system ISN–β-cyclodextrin–alginate–chitosan particles was studied by using a VEGA TESCAN TS 5130 MM scanning electron microscope (SEM). FTIR spectra of the systems and of the individual compounds were obtained from KBr pellets and collected with a PerkinElmer spectrometer „Spectrum 100 FT-IR” in the spectral range of 4000 to 650 cm^−1^ with a resolution of 1 cm^−1^.

#### Molecular docking calculations

In order to predict the binding mode and affinity of isoconazole to InhA, molecular docking was carried out. The 3D crystallographic structure of the InhA was retrieved from the RCSB Protein Data Bank (http://www.rcsb.org/pdb/), under the accession code 4U0J [28]. Before the molecular docking, the geometry of the initial structure of isoconazole was built and optimized by using the Gaussian 09 software [38]. Geometry optimization was performed using density functional theory (DFT) at the B3LYP (Becke, three-parameter, Lee-Yang-Parr)/6-31G (d, p) level [39–40].

For the docking studies, MOE (Molecular Operating Environment) [41] software was used to estimate the free energies of the enzyme–ligand binding. The enzyme–ligand complex was minimized to a gradient of 0.01 kcal/(mol·Å), and hydrogens were added using the AMBER99 force field. Charges on the enzyme and ligand were assigned using the force field AMBER99 and MMF94X, respectively. Triangle Matcher Algorithm and two rescoring functions, London dG and GBVI/WSA dG were used to produce 20 poses of isoconazole. All poses generated with docking were analyzed and the best-scoring pose for isoconazole was selected for further investigation of interactions with the enzyme InhA.
